# The Management of Orthostatic Hypotension in Parkinson’s Disease

**DOI:** 10.3389/fneur.2013.00064

**Published:** 2013-06-10

**Authors:** Álvaro Sánchez-Ferro, Julián Benito-León, Juan Carlos Gómez-Esteban

**Affiliations:** ^1^Department of Neurology, University Hospital “12 de Octubre,”Madrid, Spain; ^2^Department of Medicine, Faculty of Medicine, Complutense University, Madrid, Spain; ^3^Centro de Investigación Biomédica en Red sobre Enfermedades Neurodegenerativas, Madrid, Spain; ^4^Instituto de Salud Carlos III, Madrid, Spain; ^5^Department of Neurology, Cruces Hospital, University of the Basque Country, Bilbao, Spain

**Keywords:** treatment, orthostatic hypotension, dopamine agonists, L-DOPA, continuous dopaminergic stimulation, Parkinson’s disease

## Abstract

Orthostatic hypotension (OH) is a common and disabling symptom affecting Parkinson’s disease (PD) patients. We present the effect of the different therapies commonly used to manage PD on this clinical manifestation. For this purpose, we describe the relationship between OH and the current treatments employed in PD, such as L-DOPA, dopaminergic agonists, and continuous dopaminergic stimulation therapies. Additionally, we review the therapeutic measures that could be used to ameliorate OH. There are different approaches to deal with this manifestation, including pharmacological and non-pharmacological treatments, although none of them is specifically aimed for treating OH in PD.

## Introduction

After the first comprehensive description of the cardinal features of Parkinson’s disease (PD) (Parkinson, 2002) many non-motor features have been recognized (Hughes et al., 1992). One of the most disabling is orthostatic hypotension (OH), which is defined by a sustained reduction of systolic blood pressure (SBP) of at least 20 mmHg or diastolic blood pressure (DBP) of 10 mmHg within 3 min of standing or head-up tilt to at least 60 on a tilt table (Kaufmann, 1996; Freeman et al., 2011).

The presumed pathophysiology of OH is related to the impairment of sympathetic vasomotor neurons due to the neurodegenerative process of PD (Senard et al., 2001; Metzler et al., 2012).

Growing attention has been paid to this non-motor feature of the disease; especially since dopaminergic drugs were introduced [l-DOPA and dopamine agonists (DA)] (Barbeau et al., 1969; Calne et al., 1970; McDowell and Lee, 1970; Duby et al., 1972).

Even in those cross-sectional initial descriptions, the frequency of this symptom has been highlighted, affecting more than 30% of the PD patients (Barbeau, 1969). A similar pooled estimated prevalence has recently been reported in a systematic review (Velseboer et al., 2011). Other inferences have showed higher frequencies [41% (Kaufmann and Goldstein, 2007) and 47% in a community-based study (Allcock et al., 2004)].

Additionally, its consequences are important as it may imply an increased mortality and comorbidity (mainly owing to falls and concomitant vascular disorders) (Fedorowski et al., 2010; Maule et al., 2012).

In the current article, our aim is to review all the therapeutic options available to treat this frequent and incapacitating symptom, focusing on those aspects less studied. We begin describing the influence that common drugs and advanced therapies used in PD may exert on it. Thereafter, we detail non-pharmacological approaches that could be employed to ameliorate this symptom. We end summarizing the existing pharmacological options to treat it.

## The Effect of the Anti-Parkinsonian Therapies on Orthostatic Hypotension

One of the initial steps required when assessing a patient who is going to receive any anti-parkinsonian medication/treatment or who needs a dose adjustment is to anticipate the potential effect on his blood pressure, as it is one of the commonly related factors to the appearance of OH (Perez-Lloret et al., 2012).

Several actions could help to improve the detection of this complication, either by the physician or the subject. It seems reasonable to instruct the patient about OH symptoms (lightheadedness or dizziness after standing, fatigue, and others), although many cases go unnoticed (Arbogast et al., 2009). An initial pressor response assessment (blood pressure after standing) could be valuable to have a simple measurement to monitor future changes, as this measurement is one of the easiest ways to appraise OH in various healthcare settings.

Many caveats should be considered prior to establishing the real influence of PD medications on OH. First, there are different diagnostic criteria for defining OH. Additionally, much evidence is based on cross-sectional analyses and other confounding effects, as disease duration or previous autonomous nervous system damage (Awerbuch and Sandyk, 1992; Muller et al., 2011), have not always been considered.

We present the current evidence to estimate the potential role of current PD treatments on OH. The influence of other drugs, such as antidepressants, diuretics, and antihypertensives, is not reviewed here. Nevertheless, they should be considered when dealing with this complication and decreasing the dose or stopping the responsible medication might be advisable.

### L-DOPA

Orthostatic hypotension has been documented as a potential side effect of l-DOPA therapy since its early use on PD (Barbeau, 1969). Multiple evidence (Barbeau, 1969; Calne et al., 1970; Keenan, 1970; McDowell and Lee, 1970; Goldberg and Whitsett, 1971; Hoehn, 1975; Camerlingo et al., 1990; Senard et al., 1997; Bouhaddi et al., 2004) has supported this relationship with ranges of decline oscillating from 4.6–20 mmHg in SBP to 2.1–5.0 mmHg in DBP. Other studies failed to show any clear direct relationship (Sachs et al., 1985; Goetz et al., 1986; Haapaniemi et al., 2000; Perez-Lloret et al., 2012).

The size of the effect in the studies where a decrease on blood pressure was measured is summarized in Table 1. Methodologically limited earlier studies (small sample, not randomized, without blinding) suggested the role of fludrocortisone (0.05–0.2 mg/q.d.) and etilefrine (5 mg/t.i.d.) for treating l-DOPA-associated OH (Miller et al., 1973; Hoehn, 1975).

**Table 1:** ***Lowering effect* of PD treatments on blood pressure**.

	Reference	SBP change	DBP change	Sample size
**l-DOPA**	Bouhaddi et al. (2004)	8.1	5.0	18
	Camerlingo et al. (1990)	9.2^1^	2.1^1^	12
	Barbeau et al. (1969)	>20^2^	Na	86
	Calne et al. (1970)	8.7	4.4	20
**Dopamine agonists (excluding apomorphine)**	Haapaniemi et al. (2000)	16.1^3^	2.8^3^	17
**Monoamine Oxidase inhibitors**	Churchyard et al. (1999)	19^4^	5^4^	20
	Haapaniemi et al. (2000)	12.5^3^	5.2^3^	17
**CDS therapies**
DBS	Stemper et al. (2006)	17.7^5^	−2^5^	14
Apo	Pahwa et al. (2007b)	8.7^6^	Not reported	56
CDLI	Pursiainen et al. (2012)	23.2	9.1	9

CDS, continuous dopaminergic stimulation; DBS, deep brain stimulation; CDLI, continuous duodenal l-DOPA infusion; Apo, apomorphine pump; BP, blood pressure; SBP, systolic blood pressure; DBP, diastolic blood pressure; *values after tilting in mmHg (positive figures indicate a decrease); ^1^BP 3 min after tilting and 2 year after starting l-DOPA therapy; ^2^mean decrease after l-DOPA therapy; ^3^the blood-pressure reported is immediately after tilting; ^4^the blood-pressure reported is 2 min after tilting; ^5^blood-pressure reported after tilting with stimulation on; ^6^20 min after apomorphine injection on standing.

### Dopamine agonists

Likewise with l-DOPA, DA use has been commonly reported to produce OH in PD (Guttman, 1997; Senard et al., 1997; Lieberman et al., 1998; Korczyn et al., 1999; Pinter et al., 1999; Kujawa et al., 2000; Brunt et al., 2002; Boehringer Ingelheim Pharmaceuticals, 2003; GlaxoSmithKline, 2003a,b; UCB, Inc, 2003; Poewe et al., 2007), although not always symptomatic (Kujawa et al., 2000); however, in some studies this relationship was not shown (Hubble et al., 1995; Perez-Lloret et al., 2012).

The figures of patients treated with DA having OH are similar to that reported for l-DOPA (Stowe et al., 2008). With active standing, 34% of the DA resulted in OH (Kujawa et al., 2000); nonetheless, OH symptomatic cases listed in clinical trials ranged from 4.6 to 44.0% of the participants (Guttman, 1997; Lieberman et al., 1998; Pinter et al., 1999; Brunt et al., 2002; Boehringer Ingelheim Pharmaceuticals, 2003; GlaxoSmithKline, 2003a,b; UCB, Inc, 2003). The mean decrease of blood pressure was 16.1 mmHg in the SBP and 2.8 mmHg in the DBP (see Table 1) (Haapaniemi et al., 2000).

There is not an established causal relationship with a specific agonist, as OH has been reported in users of ergotic and non-ergotic compounds (Guttman, 1997; Korczyn et al., 1999; Haapaniemi et al., 2000; Kujawa et al., 2000; Korchounov et al., 2004; Stowe et al., 2008), and in all the current pharmaceutical preparations (conventional, extended release, and patch prescription) (Boehringer Ingelheim Pharmaceuticals, 2003; GlaxoSmithKline, 2003a,b; UCB, Inc, 2003; Poewe et al., 2007). There is no direct comparison between all DA and many clinical trials have not reported this adverse event (Giladi et al., 2007; Pahwa et al., 2007a; Stocchi et al., 2008; Stowe et al., 2008; Poewe et al., 2011; Schapira et al., 2011), so no clear conclusions can be drawn on this issue. Additionally, studies that evaluated different agonists yielded heterogeneous results. In a research involving bromocriptine, ropinirole, selegiline, l-DOPA, and amantadine, an increased frequency of OH was observed when therapies were combined (l-DOPA plus another DA) and with all the DA (a slightly greater increase was found with bromocriptine compared with ropinirole) (Korchounov et al., 2004). In meta-analytic data addressing the profile of adverse events of DA found no differences between them compared with l-DOPA (Etminan et al., 2003) and only in another meta-analytic study, an increased risk of OH was suggested for cabergoline (Kulisevsky and Pagonabarraga, 2010). In summary, no clear conclusions can be drawn concerning the risk of OH with a specific DA.

### Monoamine oxidase inhibitors

Drugs that inhibit MAO-B selectively, as rasagiline and selegiline, have been also recognized as a potential factor for inducing OH (Churchyard et al., 1997, 1999; Turkka et al., 1997; Haapaniemi et al., 2000; Bhattacharya et al., 2003; Abassi et al., 2004; Rascol et al., 2005; Olanow et al., 2009; Tolosa and Stern, 2012). There have been several studies evaluating the effect of selegiline (Churchyard et al., 1997, 1999; Turkka et al., 1997; Bhattacharya et al., 2003; Korchounov et al., 2004). The percentage of affected individuals ranged from 30% (Churchyard et al., 1999) to 50% (Bhattacharya et al., 2003) and the mean decrease oscillated between 10.4 and 19 mmHg (Churchyard et al., 1999; Bhattacharya et al., 2003) for SBP and five to even an increase of 2 mmHg for DBP (Churchyard et al., 1999; Bhattacharya et al., 2003) (Table 1). As with the previously reviewed treatments, the hypotensive effect observed with selegiline was not greater than the one occurring with other drugs such as l-DOPA (Bhattacharya et al., 2003) or DA (Haapaniemi et al., 2000) except in one study (Korchounov et al., 2004). There was an initial concern about an increased mortality associated to selegiline, also suggesting a role of its autonomic adverse effects on it, but this has been recently discarded (Lees, 1995; Ives et al., 2004; Turnbull et al., 2005). In addition to OH, hypertensive crises have been reported with selegiline (Rose et al., 2000; Ito et al., 2001).

Rasagiline is a more recent IMAO-B widely used in the clinical practice for its potential neuroprotective effect (Abassi et al., 2004; Parkinson Study Group, 2004, 2005; Rascol et al., 2005, 2011; Olanow et al., 2009; Tolosa and Stern, 2012). The percentage of individuals with symptomatic OH was comprised of between 1.5 and 6.5% (Olanow et al., 2009; Tolosa and Stern, 2012). Some of the clinical trials involving rasagiline did not make any comment about the percentage of subjects with OH and the mean decrease on blood pressure was not reported (Parkinson Study Group, 2005).

### Other common prescribed agents (L-DOPA metabolism inhibitors, anticholinergics, amantadine, acetylcholinesterase inhibitors)

There are descriptions of OH occurring with other medications such as amantadine (Korchounov et al., 2004; Perez-Lloret et al., 2012), and acetylcholinesterase inhibitors (in PD dementia) (Novartis Pharmaceuticals, 2003b). The evidence with anticholinergics is less clear, not increasing OH frequency (Martin et al., 1974), but affecting cardiovascular reflexes (Korchounov et al., 2004). The COMT inhibitors entacapone (Lyytinen et al., 2001; Novartis Pharmaceuticals, 2003a; Olanow et al., 2004) and tolcapone (Tolcapone Study Group, 1999; Koller et al., 2001), as well as DOPA-decarboxylase inhibitors, showed no clear influence on OH (Leibowitz and Lieberman, 1975; Rappelli et al., 1978), except in one study where entacapone showed a protective effect (Perez-Lloret et al., 2012).

### Is there a dose-dependent effect or an influence of therapies combinations on OH? When does OH occur in treated subjects?

It seems plausible, based on different observational approaches, that higher doses of dopaminergic medications (Senard et al., 1997; Allcock et al., 2006; Chitsaz et al., 2007) and combined therapies (Korchounov et al., 2004) could also increase the chances of manifesting OH. Also some works have suggested that the main effect of medications could be at the beginning of the therapy developing some tolerance thereafter (Pathak and Senard, 2004).

Based on all these evidence, the possibility of OH should be especially considered, when starting/adding a new drug or increasing its dose as the probability of symptoms could increase.

### Continuous dopaminergic stimulation therapies

#### Deep brain stimulation

Cross-sectional studies have suggested a positive effect of subthalamic Deep Brain Stimulation (DBS) on autonomous responses of PD subjects (Stemper et al., 2006; Ludwig et al., 2007). In one of this analysis, including 14 patients, there was a mean general decrease on blood pressure in *on* and *off* stimulation status (Table 1), but the baroreflex responses were preserved only when the stimulation was *on*, suggesting, therefore, a positive influence of the DBS in BP mediated by its influence on central autonomous nervous system pathways (Stemper et al., 2006). In another study comparing subthalamic DBS with a pharmacotherapy-only group, no positive correlation was found between the *on-stimulation* state and the decrease in blood pressure; but this occurred in the only medicated group. Based on this finding it was suggested that subthalamic DBS did not affect cardiovascular autonomous responses (Ludwig et al., 2007). Noteworthy, in a previous longitudinal study, the initial differences of blood pressure were not found after 1 year’s follow-up, with a similar mean blood-pressure decrease for the subthalamic DBS and the only medicated groups (Holmberg et al., 2005). Additionally, two other studies could not find differences in the cardiovascular responses of the treated subjects (Lipp et al., 2005; Erola et al., 2006).

In summary, subthalamic DBS could exert a neutral/positive influence at the beginning of the therapy because of its direct effects on central pathways, controlling autonomous responses (Benedetti et al., 2004), or the accompanying decrease in medication to subthalamic stimulation (Borgohain et al., 2012). This effect seems to vanish with time (Holmberg et al., 2005). In addition, medial electrode placement in the subthalamus can produce hypertensive crisis (authors experience; unpublished data). As with the common prescribed drugs, further studies will help to clarify the effect of DBS on this disabling symptom.

#### Apomorphine pump/apomorphine injections

Orthostatic hypotension has been reported since the early use of apomorphine (Duby et al., 1972; Corsini et al., 1979). In these initial descriptions, it was suggested that the peripheral DA domperidone, could diminish this complication (Corsini et al., 1979) recommending to pretreat patients 72 h before its administration. A recent report with another peripheral blocker, commercialized in the US, did not show this protective effect and only younger age influenced the development of OH in apomorphine users (Ondo et al., 2012).

The frequency of OH after apomorphine treatment is heterogeneous across the studies. The variability could be influenced, as with the other treatments, by the definitions used (manometric vs. symptomatic), pressure cutoffs, and subsets of patients evaluated. The figures oscillated between 1.9% (Tyne et al., 2004) and more than 80% of the subjects affected (Duby et al., 1972). In more recent reports, using current diagnostic criteria, a maximum of 17.6% of the subjects receiving 4 mg of apomorphine had OH vs. 14.3% of the orally treated ones (no equivalent levodopa doses reported). No clear differences between the two groups were found (Pahwa et al., 2007b). The main decrease in blood pressure was observed 20–40 min after the injection (Table 1).

#### Continuous duodenal L-DOPA infusions

There are different evidences connecting continuous duodenal l-DOPA infusions (CDLI) to OH (Pursiainen et al., 2012; Fernandez et al., 2013). In a recent clinical trial interim analysis (NCT00335153), 8.3% of the subjects had OH as an adverse event related to this therapy (Fernandez et al., 2013). In a longitudinal study involving nine CDLI cases an initial decrease of blood pressure after the therapy instauration was observed (Table 1), but after 2 months the figures rose again, suggesting a compensatory mechanism (Pursiainen et al., 2012).

### Conclusions about the effect of PD therapies on OH

Many of the common prescribed treatments used for PD could increase the frequency of OH. It seems advisable to monitor blood pressure and this side effect when starting any “*conventional medication*” or advanced PD therapy and when a dose adjustment is required. If symptoms occur adjunctive therapies should be initiated (see non-pharmacological and pharmacological treatments sections).

Other outcomes, like supine nocturnal hypertension (nocturnal BP means>120/70 mmHg) (Perez-Lloret et al., 2008), should be addressed, as patients taking higher doses of dopaminergic treatment had less decreases in SBP and DBP at night (Berganzo et al., 2013).

Further studies are needed to clarify the adjusted effect of the medication/treatments compared with the one produced by the neurodegenerative process itself.

This will help to draw more specific conclusions for subsets of subjects/treatments and anticipate the risk of OH, granting a more individualized approach when treating PD patients.

## Therapeutic Measures

### General considerations

The common practice nowadays is to manage only the symptomatic cases, as no current therapy has yet evidenced a protective action on the autonomic nervous system impairment and the role of asymptomatic OH is still not defined (Low and Singer, 2008).

It should be stressed that there are not specifically designed therapies for OH in PD subjects. This is linked to the methodological concerns limiting most of the studies presented and making the current evidence insufficient to define clear guidelines for the management of OH in PD. Nevertheless, there are different therapies that might be helpful (Lahrmann et al., 2006; Figueroa et al., 2010; Zesiewicz et al., 2010; Seppi et al., 2011), which we will review after this section.

An additional important feature to consider is that situations of orthostatic stress (early hours of the morning, meals, physical activity among others) may trigger OH that otherwise may go unnoticed (Low and Singer, 2008). Supine hypertension, an interrelated aspect of OH defined as BP means>120/70 mmHg (Perez-Lloret et al., 2008), should be also monitored (Low and Singer, 2008; Berganzo et al., 2013) as treatments used to increase blood pressure, could lead to a worsening of this manifestation. Some authors suggest that supine blood pressure should never exceed 180/110 mmHg (Low and Singer, 2008). In the case of hypertensive patients, short half-life drugs are preferable and evening administration.

Initially, it is also agreed to start with non-pharmacological measures (Lahrmann et al., 2006), because of the lower likelihood of adverse outcomes, the possibility of using them in moments of orthostatic stress (Low and Singer, 2008) and for their simplicity (Seppi et al., 2011).

### Non-pharmacological measures

In Table 2 there is a description of the main employed strategies.

**Table 2:** **Non-pharmacological therapies**.

Measure	Increase on blood pressure (mmHg)	% Compliance^1^	Comment
Fluid (water) intake	15–25	88	Recommended daily intake 2–2.5 l
Salt Intake	10–15	82	150–200 Na+ mmol/day (Salt: 9–12 g/day)
Meal frequency	Unknown	82	Multiple smaller meals containing less carbohydrates
Alcohol consumption	Unknown	59	Avoid its consume throughout the day
Night time head-up tilt	2–11	76	Elevated head of the bed (10–15 cm or 12°)
Stockings/abdominal bandages	12–26	59	Abdominal bandage more effective than stockings alone and may favor compliance
Physical countermanouvers^2^	10–15	Unknown	Leg crossing, squatting, bending-forward, and tiptoeing

*^1^Based on Schoffer et al. (2007); ^2^leg crossing, thigh contraction, or squatting*.

#### Water and salt

Standing-up implies that 500–700 ml of blood will pool in lower extremities and abdomen (Diedrich and Biaggioni, 2004). This is one of the reasons for trying to increase plasma volume to counter this effect. Many studies have evaluated the influence of drinking water on blood pressure (Jordan et al., 1999a; Senard et al., 1999; Shannon et al., 2002; Mathias and Young, 2004; Waters et al., 2005; Deguchi et al., 2007; Schoffer et al., 2007). A positive effect has been found in autonomic disorders, including multiple system atrophy or pure autonomic failure (Jordan et al., 1999a; Shannon et al., 2002; Mathias and Young, 2004; Waters et al., 2005; Deguchi et al., 2007). In PD subjects, no difference in BP has been clearly evidenced, but the sample of evaluated individuals was small (Senard et al., 1999; Schoffer et al., 2007). In non-PD-related autonomic failure, blood-pressure augmented once drinking 350–500 ml of water shortly after the ingestion (20–35 min) with a mean increase ranging between 23 and 31 mmHg for SBP and 15–25 in DBP (Shannon et al., 2002; Mathias and Young, 2004; Deguchi et al., 2007). This increase is even comparable to the one obtained with some of the commonly prescribed medications for OH (Ten Harkel et al., 1994) but lasted shortly (1 h) (Jordan et al., 1999a). It has been recommended to apply this strategy in the morning period, where blood pressure is even lower (Omboni et al., 2001; Wieling et al., 2002). The adverse outcomes are said to be mild (Schoffer et al., 2007), i.e., urinary incontinence. No follow-up data for the long-term effect have been presented (Mathias and Young, 2004).

Salt ingestion could also increase plasma volume (Wieling et al., 2002; Waters et al., 2005). One of the reasons to supplement it, is that subjects with autonomic failure are unable to reduce renal sodium excretion during salt restrictions, which could potentially lead to an increase in the blood-pressure drop upon standing (Wieling et al., 2002). The daily dietary average intake of sodium is 150 mmol (Hollenberg, 2006). An increase on blood pressure of 10–15 mmHg could be achieved with a high-salt containing diet (150–200 mmol/day of sodium or 9–12 g/day of common salt), combined with other measures (Wieling et al., 2002). It is necessary to check urine sodium (range between 170 and 260 mmol/day) (Wieling et al., 2002), as well as blood pressure (Hollenberg, 2006), to monitor the positive effects and prevent any deleterious outcomes, as high-salt intake could increase cardiovascular mortality (with urine sodium levels above 300 mmol/day) (O’Donnell et al., 2011). One study including PD cases failed to show this positive influence (Schoffer et al., 2007) after common salt supplements of 10–20 g/day (170–350 mmol/day of sodium). Another aspect yet to be determined is the salt-sensitivity of PD subjects affected by OH, as it is known that is not the same in all individuals (Hollenberg, 2006); further analyses are needed to clarify salt influence on OH in PD.

#### Physical countermaneuvers

Several drills aimed to promote venous return and maintain cardiac output have been proposed for different autonomic disorders (Ten Harkel et al., 1992, 1994; Wieling et al., 1993; Bouvette et al., 1996; Tutaj et al., 2006). They include tiptoeing, leg crossing, bending-forward, and squatting (Ten Harkel et al., 1992, 1994; Wieling et al., 1993; Bouvette et al., 1996; Tutaj et al., 2006). The range of increase in blood-pressure fluctuated between 10 and 15 mmHg (Wieling et al., 1993). The importance of these maneuvers is still controversial (Ten Harkel et al., 1994; Bouvette et al., 1996; Tutaj et al., 2006).

#### Stockings and abdominal bands

Besides the previous active maneuvers, there have been trials to raise the peripheral vascular pressure passively based on studies evaluating antigravity suits (Denq et al., 1997). This was aimed to oppose the mentioned blood pooling upon standing, exerting pressure on different capacitance beds such as the lower extremities and abdomen (Denq et al., 1997). An abdominal band has shown to be more effective and maybe with a better compliance, than the usual recommended stockings (Schoffer et al., 2007). This band could increase the blood pressure as much as 12 mmHg (Denq et al., 1997; Tanaka et al., 1997). In a study including PD patients 30 mmHg pressure stockings failed to show any beneficial effect on OH subjects (Schoffer et al., 2007).

#### Head-of-bed elevation

There is a physiological drop in blood pressure in the morning, which has been related with night recumbence, although other factors have not been ruled out (Omboni et al., 2001). In patients with PD and autonomic failure, nocturnal hypertension leads to natriuresis and polyuria, which, in turn, may cause severe OH in the morning hours. Raising the head of the bed (10–15 cm or 12°) decreased the nocturnal blood-pressure levels and the release of atrial natriuretic peptide, reducing nocturia, and OH in the early hours of the morning, especially when combined with other measures (Ten Harkel et al., 1992; van Lieshout et al., 2000). The main increase in BP in non-PD cases ranged from 2 to 11 mmHg and symptomatic relief has also been reported (Ten Harkel et al., 1992).

#### Other proposed measures

Other proposed strategies are based on evidences of different factors, which influence blood pressure in autonomic failure (Lahrmann et al., 2006; Freeman, 2008; Gupta and Nair, 2008; Low and Singer, 2008; Mostile and Jankovic, 2009). Frequent meals with fewer carbohydrates could diminish the postprandial hypotension component (Thomaides et al., 1993). It has been suggested that food could lower blood pressure in autonomic failure through vasodilatation (Thomaides et al., 1993; Chaudhuri et al., 1997) and insulin secretion (Nozaki et al., 1993). Avoiding alcohol during the day has been suggested (Chaudhuri et al., 1995; Narkiewicz et al., 2000). Averting rapid postural changes and warm temperatures and an adequate physical activity have been recommended (Lahrmann et al., 2006; Freeman, 2008; Gupta and Nair, 2008; Mostile and Jankovic, 2009). None of them have shown a favorable influence, when evaluated in PD individuals suffering from OH (Schoffer et al., 2007).

### Pharmacological measures

When non-pharmacological therapies are not satisfactory, pharmacological should be considered. Supine hypertension should be always monitored, as some of the pharmacological therapies may worsen it. Additionally, the long-term prognostic implications of OH are not known, so the aim of the therapy focuses on ameliorating the symptoms. The main drugs used to treat OH, not limited to PD, are summarized in Table 3. We describe the most important ones.

**Table 3:** **Pharmacological therapies**.

Drug	Dose	Posology	Increase on SBP/DBP (mmHg)^1^	Adverse effects	Comment
Pyridostigmine	30–60 mg	b.i.d./t.i.d.	Unknown/6.8	Abdominal colic, nausea, sialorrhea	No supine hypertension
Fludrocortisone	0.1–0.2 mg/day	q.d.	9–42/0–16	Hypokaliemia, edema, congestive heart failure, supine hypertension	Effect may appear after 1–2 weeks of treatment. Titer slowly
Midodrine	2.5–10 mg	b.i.d./t.i.d.	20–22/11–15	Paresthesia, pruritus, piloerection, supine hypertension	Tested in a randomized clinical trial
L-DOPS	200–400 mg	q.d/b.i.d.	23–28/9–12	Dizziness, tiredness, visual disturbance, supine hypertension, malignant neuroleptic syndrome	High doses of DOPA-decarboxylase inhibitors could abolish its effect
Octreotide	50–100 μg	q.d.	No differences	Injection-related, abdominal colic, tiredness, headache	Useful for postprandial hypotension
Yohimbine	5 mg	q.d	18–33/11–16	Nausea, tremor, confusion, nervousness	May be useful in postprandial orthostatic hypotension
Erythropoietin	25–75 UI/kg	3 times/week	16–19/16–17	Supine hypertension	Only if concomitant anemia is present

*SBP, systolic blood pressure; DBP, diastolic blood pressure; ^1^values are rounded*.

#### Fludrocortisone

Several studies have evaluated the effects of fludrocortisone on OH based on its volume expanding (by means of enhancing renal sodium reabsorption) and α-adrenoreceptor sensitizing activities (Hoehn, 1975; Ten Harkel et al., 1992; Schoffer et al., 2007). The common prescribed dosages range between 0.1 and 0.2 mg/day (Low and Singer, 2008). Supine hypertension could worsen under this treatment and other adverse outcomes like hypokalemia and peripheral edema should be considered (Low and Singer, 2008). The combining action with other non-pharmacological measures like salt ingestion and head-up tilt has proved to be more effective on increasing blood pressure (Ten Harkel et al., 1992).

#### Pyridostigmine

Pyridostigmine acts mainly improving ganglionic cholinergic transmissions through cholinesterase inhibition. It favors normal physiologic responses upon standing, without worsening supine hypertension (Singer et al., 2006; Low and Singer, 2008). The pressor effect was modest (a 6.4 mmHg increase in DBP) (Singer et al., 2006), so it is recommended for initial treatment or mild cases (Low and Singer, 2008). Doses are started at 30 mg b.i.d. or t.i.d. and could be increased to 60 mg b.i.d. or t.i.d (Singer et al., 2006). Adverse outcomes include diarrhea and nausea, among other cholinergic symptoms (Singer et al., 2006; Low and Singer, 2008).

#### Midodrine

This drug is a α1-agonist with a short duration effect (2–4 h). Its main action is to augment vascular resistance and therefore blood pressure. In addition to the anti-OH effect, it can also enhance supine hypertension, so close monitoring of recumbent blood pressure and avoiding evening administration are required (Jankovic et al., 1993; Low and Singer, 2008). Doses range between 2.5 and 10 mg b.i.d or t.i.d (Jankovic et al., 1993; Low et al., 1997). Along with hypertension, other common adverse events are piloerection, paresthesia, and itching (characteristically in the scalp) (Jankovic et al., 1993; Low et al., 1997).

#### L-DOPS

l-Threo-dihydroxyphenylserine or droxidopa is a pro-drug converted by DOPA decarboxylase into norepinephrine. Doses between 100 and 900 mg have been used in PD and other autonomic disorders, improving the drop in blood pressure in OH significantly (Kaufmann, 2008; Mathias, 2008). Side effects were mild, but supine hypertension should be monitored (Kaufmann, 2008). Noteworthy, DOPA-decarboxylase inhibitors used in high doses (200 mg) could block this therapeutic effect (Kaufmann, 2008), but the current combinations employed with l-DOPA (25–50 mg of inhibitor) do not produce this interference (Kaufmann, 2008). Recently, promising results suggesting a positive influence on symptoms and OH related outcomes (falls and fall related injuries) have been presented by Isaacson et al. (2013), American Academy of Neurology, San Diego, CA, USA.

#### Other drugs

Many other medicaments have been attempted to correct the effects of OH. The evidence in which they are based is weak, mostly because of the small samples sizes and the designs used. We describe some, but this is not intended to be an exhaustive review of them.

Octreotide has been used mainly to correct postprandial hypotension linked to OH (Hoeldtke and Israel, 1989), as it could counter the release of vasoactive peptides secreted with food/alcohol ingestion (Bordet et al., 1995; Chaudhuri et al., 1995; Hoeldtke et al., 1998). Its combination with midodrine was more effective than when it was given alone (Hoeldtke and Israel, 1989). The doses range from 50 to 100 μg/kg (subcutaneous injection). In some studies, no clear change of blood pressure could be evidenced, but symptomatic relief was reported (Bordet et al., 1995).

Yohimbine is an alpha-2 adrenergic receptor antagonist, which enhances the residual sympathetic tone (Onrot et al., 1987; Shibao et al., 2010). Some reports, including PD cases, have suggested a positive role and even better results on raising blood pressure than pyridostigmine (Shibao et al., 2010).

Erythropoietin has been useful in correcting a drop in blood pressure in patients with concomitant anemia and autonomic failure (Hoeldtke and Streeten, 1993; Perera et al., 1995).

Desmopressin studies have suggested a positive influence on orthostatic tolerance (Mathias et al., 1986; Larina et al., 2009). Desmopressin reduced nocturnal polyuria, raised supine blood pressure, and reduced the postural fall, especially in the morning, when patients were often at their worst (Mathias et al., 1986; Larina et al., 2009).

Caffeine has been studied alone or in combination with ergotamine to treat OH and postprandial hypotension in subjects with PD and other conditions causing autonomic failure (Lipsitz et al., 1994; Dewey et al., 1998).

Domperidone (30 mg t.i.d), a dopaminergic antagonist, has also shown beneficial effects on OH (Montastruc et al., 1985; Lang, 2001; Schoffer et al., 2007). It has also been recommended to treat autonomic symptoms related to apomorphine (see above).

Ephedrine is an indirect sympathomimetic agent that has also been used in OH (Brooks et al., 1989), but due to its central nervous system actions, midodrine, that does not cross the blood-brain barrier, may be preferred. Supine hypertension is also related to its use (Brooks et al., 1989).

Etilefrine (5–10 mg) was used in 15 PD patients to increase blood pressure upon standing (mean increase of 4.3%) reporting only headaches as adverse outcomes (Miller et al., 1973).

Fluoxetine (20 mg) was used in a pilot study including 14 PD patients, reducing the drop in blood pressure in 11 mmHg and improving orthostatic symptoms (Montastruc et al., 1998).

For treating supine hypertension, it has been recommended to use nitroglycerine patches (0.025–0.2 mg/h) (Jordan et al., 1999b), or clonidine (0.1 mg) (Shibao et al., 2006), but further studies are needed to measure the impact of this drugs on PD cases.

## Overall Summary

Orthostatic hypotension is a common and challenging symptom affecting PD patients. The neurodegenerative process is responsible for damaging the autonomous nervous system, but anti-parkinsonian treatments could enhance the symptoms derived from it. Current therapeutical strategies include non-pharmacological and pharmacological measures aimed to favor baropressor responses or to increase blood volume. There is insufficient evidence to recommend any specific treatment for the PD-related autonomic failure. Therefore, it should be individualized for the individual patient. Studies addressing the underscored questions related to OH in PD are needed.

## Conflict of Interest Statement

The authors declare that the research was conducted in the absence of any commercial or financial relationships that could be construed as a potential conflict of interest.
